# Obstructive Sleep Apnea in a Clinical Population: Prevalence, Predictive Factors, and Clinical Characteristics of Patients Referred to a Sleep Center in Mongolia

**DOI:** 10.3390/ijerph182212032

**Published:** 2021-11-16

**Authors:** Shuren Dashzeveg, Yasunori Oka, Munkhjin Purevtogtokh, Enkhnaran Tumurbaatar, Battuvshin Lkhagvasuren, Otgonbayar Luvsannorov, Damdindorj Boldbaatar

**Affiliations:** 1 Department of Neurology, School of Medicine, Mongolian National University of Medical Sciences, Zorig Street 3, Sukhbaatar District, Ulaanbaatar 14210, Mongolia; dasshuren@gmail.com (S.D.); otgonbayar.l@mnums.edu.mn (O.L.); 2 Sleep Center, General Hospital for State Special Servants, Ulaanbaatar 15160, Mongolia; 3 Center for Sleep Medicine, Ehime University Hospital, Shitsukawa, Toon 791-0295, Ehime, Japan; 4 School of Medicine, Mongolian National University of Medical Sciences, Zorig Street 3, Sukhbaatar District, Ulaanbaatar 14210, Mongolia; munkhjinpurevtogtokh@gmail.com; 5 Brain Science Institute, Graduate School, Mongolian National University of Medical Sciences, Zorig Street 3, Sukhbaatar District, Ulaanbaatar 14210, Mongolia; enkhnaran@mnums.edu.mn (E.T.); battuvshin@mnums.edu.mn (B.L.); 6 Department of the Health Research, Graduate School, and Department of Physiology, School of Biomedicine, Mongolian National University of Medical Sciences, Zorig Street 3, Sukhbaatar District, Ulaanbaatar 14210, Mongolia

**Keywords:** apnea–hypopnea index, polysomnography, hospital based, anthropometric characteristics, multiple linear regression

## Abstract

Obstructive sleep apnea (OSA) disrupts sleep. This study examined factors related to OSA severity. A cross-sectional, prospective, hospital-based study was conducted with 205 patients who underwent polysomnography (PSG). Demographic, anthropometric, clinical, PSG, and sleep quality assessment data were analyzed. Participants (N = 205) were classified into four groups based on apnea–hypopnea index (AHI); no OSA (AHI < 5/h; N = 14), mild (mOSA, 5 < AHI < 15/h; N = 50), moderate (modOSA, 15 < AHI < 30/h; N = 41), severe (sOSA, 30 < AHI < 60/h; N = 50), and very severe (vsOSA, AHI ≥ 60; N = 50). Men had more severe OSA than women (*p* < 0.001). Anthropometric characteristics differed with OSA severity (*p* < 0.001). OSA patients had decreased sleep quality and increased excessive daytime sleepiness (EDS). Body mass index (BMI), neck/waist circumference, and blood pressure (BP) differed between groups (*p* < 0.001). Patients with vsOSA had the highest Mallampati grades (*p* < 0.001). Multiple linear regression indicated that OSA severity was related to gender and sleep quality. PSG parameters (oxygen saturation, systolic BP, and arousal/respiratory arousal) were strongly related to OSA severity. In conclusion, about half of study-referred patients had severe/very severe OSA; these groups were predominantly obese men with high BP. OSA severity associated with high BP, BMI, waist circumference, and neck circumference.

## 1. Introduction

Obstructive sleep apnea (OSA) is a common condition characterized by episodic hypoxia due to repetitive obstruction of the upper airway during sleep, which triggers an arousal response, thereby disrupting restful sleep. People with OSA may complain of excessive daytime sleepiness (EDS) or insomnia, nocturia, and morning headaches. OSA has been associated with numerous negative outcomes, including sleep disruption, daytime sleepiness, cognitive dysfunction, and cardiovascular disease [1]. There has been increasing concern regarding lapses in attention due to EDS, a major OSA symptom, leading to increased risk of traffic accidents and errors by workers that can cause serious industrial accidents [2,3,4]. Generally, OSA is diagnosed based on the apnea–hypopnea index (AHI), which indicates the number of apnea/hypopnea events per hour of sleep, wherein a pause in breathing lasting 10 s or longer with an associated decrease in blood oxygenation is considered an event. Five or more such apnea/hypopnea events per hour is considered indicative of OSA [1].

The prevalence of symptomatic OSA in the general population has been estimated to be 3~9%, with substantial variance between and within regions [1,5]. Global prevalence data estimate the prevalence from 3.0% up to 36.6% in different countries [6]. In Western countries, reported OSA prevalence rates range from 9% to 38% [7]. Additionally, it has been postulated that up to 5% of Western country populations may have undiagnosed OSA syndrome [8]. In Asian countries, reported OSA prevalence rates range from 3.7% to 97.3% [9]. The prevalence and characteristics of OSA in Mongolia are not yet known.

Sleep-related risk factors with strong associations include male sex, older age, and obesity; other factors with moderate associations include craniofacial/upper-airway abnormalities, smoking, alcohol consumption, nasal congestion, cardiovascular disease, and a family history of sleep apnea [1,10,11]. In the Wisconsin Sleep Cohort study involving a stratified random sample of Wisconsin state employees, 30–60 years of age, the prevalence of OSA (i.e., AHI > 5) was 9% in women and 24% in men [8]. The prevalence of OSA has been shown to increase with age in adults, at least up to the age 65 [11]. Prevalence of OSA was reported to be 88% among geriatric patients mostly with mild dementia [12]. Regarding the association of obesity with OSA, high levels of fat deposition around the upper airway are thought to increase risk of OSA. This association can be seen with high body mass index (BMI) or neck circumference. Moderate to severe OSA is often accompanied by snoring, self-reported gasping, observations of apnea by one’s sleep companion, and hypertension, in addition to a high BMI or neck circumference.

Several clinical questionnaires are used to identify patients at risk for OSA, including the STOP-BANG [13], the Epworth Sleepiness Scale (ESS) [14], the Pittsburg Sleep Quality Index (PSQI) [15], and the Functional Outcome Sleep Questionnaire (FOSQ) [16]. A conclusive diagnosis is made by polysomnography (PSG). Typically, the following clinical AHI classifications based on PSG analysis are applied: <5 events/h, no OSA; ≥5 and <15 events/h, mild OSA; ≥15 and <30 events/h, moderate OSA; and ≥30 events/h, severe OSA [17,18,19]. Additional AHI cutoff levels have been applied in clinical studies, including an AHI ≥ 60 cutoff to define very severe OSA (vsOSA) [20,21,22] and an AHI > 100 cutoff to define extreme OSA [23]. Prevalence of vsOSA is unclear, but 8.7% was reported to be in this category in a relatively small sample of OSA population [22]. These additional strata have provided additional information about how OSA severity relates to morbidity and mortality.

The aim of the present study was to characterize the demographic, clinical, and sleep characteristics of patients with OSA, based on AHI-defined severity and analyze the predictive factors for OSA. Based on a previous report [19], we hypothesized that we would find that vsOSA would be associated with greater comorbidities than are found in less severe OSA.

## 2. Materials and Methods

### 2.1. Study Design and Participants

A cross-sectional, prospective, hospital-based study was conducted. Patient data and PSG recordings from a 2-year period (November 2018 to November 2020) were collected from the Sleep Center in the Neurology Department at the General Hospital for State Special Servants in Ulaanbaatar, Mongolia (referred to as the Sleep Center from here forward). All patients were referred from primary, secondary, and tertiary hospitals and clinics for the diagnosis of sleep disorders mainly obstructive sleep apnea. A total of 233 PSG recordings were performed at the Sleep Center during the aforementioned 2-year period. All PSG were adult cases except for an adolescent case aged15 years old. After excluding 28 recordings that represented repeat PSG assessments, the remaining 205 were included in the study.

All 205 patients underwent an overnight PSG recording from 10:00 pm to 6:00 am following outpatient referrals from a sleep specialist or neurologist. In the sleep laboratory, doctors and nurses received patients for PSG between 7 pm and 8 pm, at which time they performed body measurements, checked each patient’s blood pressure (BP) and oxygenation (SpO_2_) level, asked the patient to complete self-administered sleep questionnaires, and then hooked up the PSG electrodes. Body measurements included body weight, neck circumference, waist circumference, Mallampati index, and Tonsill score. The patients each completed ESS [14], PSQI [16], FOSQ [16], and STOP-BANG [13] self-reported questionnaires.

The study did not cause any risk to patients. All patients signed informed consent forms before undergoing clinical examination, anthropometric measurements, and PSG. The institutional review board and Ethics Committee of the Mongolian National University of Medical Sciences (no. 2019/3-03, 23 March 2019, Ulaanbaatar, Mongolia) approved the study protocol.

### 2.2. Variables and Measurements

Independent and dependent variables: the independent variables included clinical and body measurements. The dependent variables included PSG parameters and questionnaire scores. OSA severity category (no, mild, moderate severe, or very severe) was a main dependent variable derived from PSG data.

Covariates and other variables: education level was categorized as primary, secondary, and high. Residency was divided by city and province; employment by unemployed, student, pensioner, employed, and disabled.

### 2.3. Clinical Examination

During the clinical examination, each participants’ body weight (in kg) and height (in cm) were measured by well-trained medical assistants or a physician, and then the participant’s BMI (in kg/m^2^) was calculated. Patients with a BMI between 25 and 30 kg/m^2^ were considered overweight, and patients with BMI values >30 kg/m^2^ were considered obese [24]. Body weight was measured with an AQUNICC BC360 body composition analyzer (2017 model, SELVAS Healthcare company, Seoul, Korea) to the nearest 0.5 kg while the patient stood barefoot and wearing light indoor clothes. Each participant’s waist circumference was measured (in cm) midway between the lower rib margin and anterior superior iliac spine with a non-elastic measuring tape. Neck circumference was measured (in cm) at the level of cricothyroid membrane. BP was determined by an automatic sphygmomanometer with a digital display (Raycome, China) with the patient sitting after being at rest for at least 5 min. The criterion for hypertension was a systolic BP >140 mmHg and/or a diastolic BP >90 mmHg.

The oropharynx was examined to determine each patient’s Mallampati score and tonsil size by asking the patient to open his or her mouth as wide as possible, while protruding the tongue as far as possible. All oropharynx assessments were performed or supervised directly by the same physician. The following standard Mallampati I to IV grading system was used: I, uvula and entire tonsils/pillars clearly visible; II, uvula mostly visible but tonsils or pillars not visible; III, only soft palate visible fully or partially; and IV, only the hard palate visible. Simultaneously, tonsil size was graded from I to IV: I, tonsillar tissue not visible; II tonsils visible within tonsillar pillars; III, tonsils protrude beyond pillars; and IV, tonsils protrude to the midline [25,26].

### 2.4. Questionnaires

#### 2.4.1. STOP-BANG

The STOP-BANG questionnaire was developed to be a reliable, concise, and easy-to-use OSA screening tool. It consists of eight dichotomous (yes/no) items related to the clinical predictors of sleep apnea: snoring, tiredness, observed apnea, high BP, BMI, age, neck circumference, and male gender. The total score ranges from 0 to 8 (1 point per yes). Scores in the range of 0–2 indicate low risk, 3–4 indicate moderate risk, and 5–8 indicate high risk for OSA [13].

#### 2.4.2. ESS

The ESS is a simple, self-administered 8-item questionnaire that provides a measurement of one’s general level of daytime sleepiness. It asks subjects to rate how likely they, in recent weeks, would be to “doze off or fall asleep” in eight situations of daily living, such as “sitting and reading” or “watching TV”. They are asked to imagine how they would respond if they have not done a given activity recently. Each item is answered on a 4-point scale: 0, would never doze; 1, slight chance of dozing; 2, moderate chance of dozing; and 3, high chance of dozing. Scores range from 0 to 24, with higher scores indicating greater daytime sleepiness. Scores ≥11 were considered abnormal, and indicative of EDS [14].

#### 2.4.3. PSQI

The PSQI is a self-rated questionnaire designed to measure sleep quality and disturbances over the prior month and to discriminate between good and poor sleepers. It consists of 7 components (19 total items): subjective sleep quality (1 item), sleep latency (2 items), sleep duration (1 item), habitual sleep efficiency (3 items), sleep disturbances (9 items), use of sleeping medications (1 item), and daytime dysfunction (2 items). Items 1–4 (asking for usual bedtime and wake up times, number of minutes to fall asleep, and hours slept per night) are free response and not included in scoring. All remaining items are answered on 4-point scales (0–3 points). Item 18 asks respondents to indicate their overall sleep quality and Item 19 asks respondents to rate their enthusiasm to get things done [15].

#### 2.4.4. FOSQ

The FOSQ is a 30-item instrument designed to assess the impact of EDS and fatigue on functional outcomes relevant to daily behaviors and sleep-related quality of life. It assesses five domains of day-to-day life: activity levels, vigilance, intimacy and sexual relationships, productivity, and social outcomes. The questionnaire is indicated for both research and clinical purposes (screening, assessing treatment outcomes, etc.) [16].

We used copyright agreements for the STOP-BANG and PSQI questionnaires given to the Brain Science Institute at the Mongolian National University of Medical Sciences and this study constitutes the completion of the first phase of a cross-cultural validation study.

### 2.5. PSG

A single all-night PSG protocol was followed. Standardized digital recordings for all participants were obtained on a single SOMNOscreenTM plus 6962 device (SOMNOmedics GmbH, Randersacker, Germany) and analyzed in DOMINO (version 2.6.0) software. PSG preparation, including placement of electrodes, took 50~60 min. PSG took place under vigilant observation by specialized technicians with the supervision of a physician, in a room with appropriate technical conditions (10:00 pm bedtime, 6:00 wake-up time). PSG recordings were conducted and scored according to the American Academy of Sleep Medicine (AASM) scoring manual (version 2.4) [27], by experienced technicians.Electroencephalograms (EEGs) were obtained from two central leads (C4-M1, C3-M2), two occipital leads (O2-M1, O1-M2), and two frontal leads (F4-M1, F3-M2). Electrooculogram (EOG), electromyogram (EMG), electrocardiogram (ECG), nasal pressure, oronasal airflow, snoring, thoracic and abdominal respiratory movements, pulse oximetry, right and left anterior tibialis EMG were recorded. Apnea was defined as a decrease in amplitude of the thermistor signal of ≥90% for at least 90% of the duration of an event lasting ≥10 s. Hypopnea was defined using the acceptable scoring criteria (rule 1B) of the AASM scoring manual; a decrease in amplitude of the nasal pressure signal of ≥30% which lasts ≥10 s with oxygen desaturation ≥4% (compared to pre-event baseline). Apnea–hypopnea index (AHI)was used to determine the severity category of OSA in this study as follows: <5 events/h (no OSA), ≥5 and <15 events/h, mild OSA (mOSA); ≥15 and <30 events/h, moderate OSA (modOSA); ≥30 events/h, severe OSA (sOSA); and ≥60 events/h, very severe OSA (vsOSA) [17,18,19].

### 2.6. Statistical Analysis

The data are presented as a means ± standard deviations (SDs). The normality of the distributions of continuous variables was determined with the Kolmogorov–Smirnov test. Differences between categorical variables were detected with Chi-square tests. One-way analyses of variance (ANOVAs) were used to detect differences in PSG characteristics and questionnaire scores across OSA severity groups. Multiple linear regression tests were performed to evaluate the association between AHI and all variables. We analyzed unstandardized regression coefficients. Data analyses were conducted in SPSS version 26.0 (IBM, New York, NY, USA) with a *p*< 0.05 criterion for significance (two-sided).

## 3. Results

### 3.1. Demographic Characteristics

The demographic characteristics of the study sample of patients who underwent PSG in the Sleep Center are presented in Table 1. The majority of the patients, overall, were male, had a higher education, and were residents of Ulaanbaatar city. Almost half were employed. Of the 205 patients in the sample, 191 (93.2%) had an AHI >5 and were thus diagnosed with OSA; the remaining 14 patients (6.8%) did not have OSA (no OSA group). The distribution of participants across AHI severity groups is shown in Table 1 together with comparisons of the demographic variables across the groups. The mean (±SD) age and age range for the whole sample was 48.7 ± 12.6 years (15–74 years). The mean ages and age range for the groups were similar to one another as follows (in years): no OSA, 43.7 ± 15.6 (15–74 years); mOSA, 48.3 ± 12.4 (22–69 years); modOSA, 50.0 ± 13.1 (18–70 years); sOSA, 51.8 ± 11.8 (27–73 years); vsOSA, 47.1 ± 11.2 (26–69 years) (*p* = 0.158).

### 3.2. Clinical Examination

The clinical examination findings for the cohort are summarized in Table 2. Based on the clinical examination findings, it was determined that 141/205 participants (68.8%) had high BP, 91 were overweight, and 100 were morbidly obese. As shown in Table 2, BMI, neck circumference, waist circumference, systolic BP, and diastolic BP differed very significantly between the OSA severity groups. Patients with vsOSA had higher Mallampati grades than patients in the other OSA severity groups (*p*< 0.001), though tonsil size did not differ significantly among the groups (Table 2).

### 3.3. Sleep Questionnaires

The sleep quality questionnaire results for the study sample and for each group are presented in Table 3. STOP-BANG, ESS, PSQI, and FOSQ scores differed significantly among the OSA severity groups. The no OSA and mOSA groups had low STOP-BANG scores, whereas the modOSA, sOSA, and vsOSA groups had increasing scores with increasing OSA severity. Likewise, ESS scores increased with increasing OSA severity, indicating that greater OSA severity is associated with more EDS. According to the FOSQ scores, only the mOSA and modOSA groups reported greater dysfunction than the no OSA group. Moreover, PSQI scores decreased with increasing OSA severity, suggesting that patients in the sOSA and vsOSA groups tended to believe they were getting good sleep.

### 3.4. PSG

There were significant differences among the OSA severity groups for all PSG parameters except total sleep time, sleep efficiency, deep (sleep) latency, and arrhythmia (Table 4). Relative to the no OSA and mOSA groups, the higher severity groups tended to have longer periods of light sleep (N1, N2) with reduced deep sleep (N3) periods, reduced REM sleep periods, and reduced SpO_2_ values, as well as higher obstructive apnea, arousal index, respiratory arousal index (RAI), snore index, Cheyne–Stokes respiration index (ChSI), and nocturnal BP values (Table 4).

### 3.5. Factors Associated with AHI

Multiple regression analysis of factorsrevealed that gender, STOPBANG score, ESS score, and PSQI score were associated with AHI severity category. Increased neck circumference, abdominal circumference, and Mallampati grade emerged as predictive factors of AHI severity. With respect to sleep study variables, decreased SpO_2_ levels, increased nocturnal systolic BP, increased arousal, and increased respiratory arousal were strongly associated with AHI severity (Table 5). The full regression analysis results are shown in the Appendix A (Table A1).

## 4. Discussion

In this study, we determined the prevalence of OSA severity class within a referred Mongolian clinical population for the first time using PSG. The data obtained in this study provide a baseline for future work on OSA screening.

In the present 2-year study, 90% of the Sleep Center-referred PSG patients had an AHI >5 and were thus diagnosed with OSA. Notably, more than half of the referred patients had sOSA or vsOSA (N = 50 each), which is high compared to other reports in the literature [28]. The present findings relating greater OSA severity with a high BMI and large abdominal circumference are consistent with the findings of several prior studies [29,30,31]. Furthermore, our findings of higher nocturnal systolic BP in vsOSA patients than in patients with moderate OSA are consistent with similar observations in several prior studies [31,32,33,34]. Moreover, our finding of hypertension being more prevalent in vsOSA patients than in patients with less severe OSA is consistent with prior reports [35,36].

In our Mongolian sample, patients with vsOSA were younger than vsOSA groups described in prior studies conducted in Western and Asian countries [9,37]. The presently observed associations of AHI severity with anthropometric measurements, including BMI, waist circumference, and neck circumference, were similar to associations reported previously [22]. Compared with other similar studies [38,39], the PSG architecture of our patients indicated higher AHI values but similar total sleep time, sleep efficiency, N3, REM, and arousal index values. Our finding of a very strong relationship of decreasing SpO_2_ during sleep with greater AHI severity in our Mongolian patients was also similar to the results of prior studies [29,30].

There are few studies that have examined the risk factors and clinical characteristics of extreme OSA (AHI > 100). During our 2-year study in Mongolia, we identified only 9 patients with an AHI > 100. A study conducted in Argentina over 1.5 years identified 10 patients with an AHI > 100 [21] and another South American study conducted over a 6-year period identified 19 patients with an AHI ˃ 100 [40]. Together with these two prior studies, our patient distribution suggests that an AHI > 100 is relatively rare across global populations. Interestingly, the associations revealed by our multiple linear regression analysis are consistent with the associations found in a prior study that was focused on patients with extreme OSA [23]. They found that AHI severity was associated consistently with male gender, larger waist and neck circumferences, high Mallampati grade, high ESS score, and high STOP-BANG scores, as well as with sleep study variables, including a high nocturnal systolic BP, high Cheyne–Stokes respiration occurrence, high snore index, high arousal index, high respiratory arousal index, and low nocturnal SpO_2_.

This study was conducted with relatively small number of patients who underwent sleep investigation in one sleep center in Mongolia, which caused sampling bias and difficulty in comparing the data with other populations/ethnicities including cranio-facial structures and socio-economic status. This study had several methodological limitations. Firstly, there are a lack of sleep questionnaires validated for use with Mongolian people. Thus, we used adopted questionnaires. Secondly, we did conduct a broad clinical laboratory analysis inclusive of blood sugar, cholesterol, and lipids, which may differ across OSA severities. Thirdly, we did not include alternative hypopnea criteria of ≥3% desaturation and/or EEG arousals which is allowed as an alternative in the AASM scoring manual [27]. As the discrepancies among hypopnea definitions used in research studies were pointed out to introduce complexity in the evaluation of evidence regardingthe diagnosis of OSA, the hypopnea criteria used could have affected the study results [18].

## 5. Conclusions

This study identified clinical characteristics of OSA in Mongolia for the first time with gold standard polysomnography. More than 9 in 10 Mongolian patients referred to our hospital-based Sleep Center for sleep studies were found to have OSA based on AHI ratings. Approximately half of the OSA diagnosed patients had sOSA or vsOSA. The sOSA and vsOSA groups were predominantly constituted by obese young men with high BP. Common characteristics of patients with sOSA or vsOSA were high BP, high BMI, a large waist circumference, and a large neck circumference. Sleep questionnaire and PSG data indicated that, relative to patients with mOSA or modOSA, patients with sOSA or vsOSA exhibited longer sleep latencies, more prolonged periods of light sleep, lesser durations of deep sleep, higher arousal index values, more Cheyne–Stokes respiration, higher nocturnal BP, and lower oxygen saturation. The association of a high arousal index, Cheyne-Stokes respiration, and nocturnal BP with OSA may represent risks for cardiovascular and neurological disorders in patients with OSA. The clinical situation may be similar in developing countries where OSA patients are undiagnosed as in Mongolia.

## Figures and Tables

**Table 1:** Demographic characteristics of patients in sample and across OSA groups.

Character	Total N = 205	OSA Group, N (%)					
		No 14 (6.8)	Mild 50 (24.4)	Moderate 41 (9.8)	Severe 50 (24.4)	Very Severe 50 (24.4)	*p*
**Age group, y**							0.112
15–19	2 (1.0)	1 (7.1)	0 (0.0)	1 (2.4)	0 (0.0)	0 (0.0)	
20–29	16 (7.8)	2 (14.3)	6 (12.0)	2 (4.9)	3 (6.0)	3 (6.0)	
30–39	37 (18.0)	2 (14.3)	11 (22.0)	9 (22.0)	4 (8.0)	11 (22.0)	
40–49	48 (23.4)	4 (28.6)	8 (16.0)	7 (17.0)	13 (26.0)	16 (32.0)	
50–59	58 (28.3)	2 (14.3)	18 (36.0)	11 (26.8)	14 (28.0)	13 (26.0)	
60–74	44 (21.5)	3 (21.4)	7 (14.0)	11 (26.8)	16 (32.0)	7 (14.0)	
Sex							<0.001
Male	105 (51.2)	4 (28.6)	12 (24.0)	16 (39.0)	28 (56.0)	45 (90.0)	
Female	100 (48.8)	10 (71.4)	38 (76.0)	25 (61.0)	22 (44.0)	5 (10.0)	
Education							
High	143 (69.8)	7 (50.0)	34 (68.0)	30 (73.2)	36 (72.0)	36 (72.0)	0.641
Secondary	43 (21.0)	6 (42.9)	10 (20.0)	8 (19.5)	9 (18.0)	10 (20.0)	
Primary	19 (9.3)	1 (7.1)	6 (12.0)	3 (7.3)	5 (10.0)	4 (8.0)	
Residency							
Ulaanbaatar	167 (81.5)	13 (92.9)	45 (90.0)	35 (85.4)	40 (80.0)	34 (68.0)	0.04
Rural areas	38 (18.5)	1 (7.1)	5 (10.0)	6 (14.6)	10 (20.0)	16 (32.0)	
Employment							
Unemployed	42 (20.5)	4 (28.6)	10 (20.0)	11 (26.8)	7 (14.0)	10 (20.0)	0.571
Student	4 (2.0)	2 (14.3)	1 (2.0)	1 (2.4)	0 (0.0)	0 (0.0)	
Retired	59 (28.8)	1 (7.1)	14 (28.0)	13 (31.7)	20 (40.0)	11 (22.0)	
Employed	97 (47.3)	7 (50.0)	25 (50.0)	15 (36.6)	21 (42.0)	29 (58.0)	
Disability	3 (1.5)	0 (0.0)	0 (0.0)	1 (2.4)	2 (4.0)	0 (0.0)	

*p*-values are from Chi-square tests.

**Table 2:** Clinical characteristics of the patient sample and across OSA severity groups.

Characteristic	Total	OSA Group, Mean ± SD or N (%)					
		No	Mild	Moderate	Severe	Very Severe	*p*
**BMI, kg/m^2^**	29.2 ± 6.34	25.5 ± 4.01	27.6 ± 4.14	30.1 ± 6.34	34.9 ± 5.7	29.2 ± 6.3	<0.001
Neck, cm	37.1 ± 5.2	33.9 ± 3.4	35.9 ± 4.2	38.1 ± 5.0	41.4 ± 4.33	37.1 ± 5.2	<0.001
Waist, cm	98.4 ± 18.1	87.6 ± 12.2	93.6 ± 12.8	102 ± 17.3	114.1 ± 16.3	98.4 ± 18.1	<0.001
Systolic BP	137.1 ± 23.3	126.9 ± 17.2	133.3 ± 23.9	140.2 ± 24.0	150.5 ± 22.7	137.1 ± 23.3	<0.001
Diastolic BP	87.7 ± 11.3	82.5 ± 11.5	88.4 ± 11.7	89.4 ± 10.6	92.0 ± 9.7	87.7 ± 11.3	0.001
Mallampati grade							
I	12 (85.7)	45 (82.4)	32 (78)	26 (52)	21 (42.0)	130 (63.4)	<0.001
II	1 (7.1)	5 (10.0)	6 (14.6)	20 (40.0)	18 (36.0)	50 (24.4)	
III	1 (7.1)	0 (0.0)	3 (7.3)	4 (8.0)	8 (16.0)	16 (7.8)	
IV	3 (1.5)	0 (0.0)	0 (0.0)	0 (0.0)	0 (0.0)	3 (6.0)	
Tonsil size							
I	11 (78.6)	35 (70.0)	26 (63.4)	26 (52.0)	25 (50.0)	123 (60.0)	0.117
II	2 (14.3)	11 (22.0)	14 (34.1)	20 (40.0)	19 (38.0)	66 (32.2)	
III	1 (7.1)	4 (8.0)	1 (2.4)	2 (4.0)	5 (10.0)	13 (6.3)	
IV	3 (1.5)	0(0.0)	0(0.0)	0(0.0)	2 (4.0)	1 (2.0)	

*p*-values from Chi-square tests or one-way ANOVA test as appropriate.

**Table 3:** Sleep questionnaire responses for the study sample and across OSA severity groups.

Scale	Total Median (IQR 25–75%)	OSA Group, Median (IQR 25–75%)					
		No	Mild	Moderate	Severe	Very Severe	*p*
STOP-BANG	3.0 (2.0–5.0)	1.0 (1.0–2.8)	1.5 (1.0–2.0)	2.0 (1.0–3.0)	4.0 (3.0–5.0)	5.0 (4.0–6.0)	<0.001
ESS	8.0 (4.0–12.0)	5.0 (0.8–9.8)	6.0 (3.3–11.8)	7.0 (4.0–11.0)	8.0 (4.2–13.0)	10.0 (6.0–12.7)	0.060
PSQI	9.0 (5.0–12.0)	5.0 (3.0–8.0)	8.0 (6.0–13.0)	10.0 (8.0–14.0)	11.0 (8.0–13.0)	13.0 (9.7–15.7)	<0.001
FOSQ	54.0 (47.0–67.0)	56.5 (48.2–68.2)	50.0 (45.0–65.0)	48.0 (45.0–62.0)	58.0 (49.0–68.7)	58.0 (49.0–67.7)	0.004

*p*-values from one-way ANOVA.Possible ranges: STOP-BANG (0–8), ESS (0–24), PSQI (0–21), and FOSQ (0–120).

**Table 4:** Polysomnography characteristics of patients with different severity of OSA.

Characteristic	Total	OSA Group, Mean ± SD					
		No	Mild	Moderate	Severe	Very Severe	*p*
AHI, /hr	37.26 ± 30.05	3.0 ± 1.5	9.7 ± 3.2	21.6 ± 4.8	43.3 ± 8.25	81.3 ± 18.2	<0.001
Total sleep time, min	6.96 ± 1.05	7.28 ± 0.82	6.85 ± 1.23	6.94 ± 1.16	6.75 ± 0.97	7.20 ± 0.83	0.180
Sleep efficiency, %	86.1 ± 10.8	87.3 ± 8.35	85.5 ± 12.7	85.1 ± 11.2	85.0 ± 10.5	88.4 ± 9.16	0.383
Sleep latency, min	14.1 ± 24.1	15.1 ± 9.04	20.0 ± 41.3	12.9 ± 15.6	12.5 ± 11.2	10.5 ± 17.4	0.024
Deep latency, min	78.9 ± 77.6	56.6 ± 58.6	77.0 ± 80.1	88.0 ± 95.4	81.0 ± 66.1	77.6 ± 75.8	0.599
Wake, %	86.1 ± 10.8	87.3 ± 8.35	85.5 ± 12.7	85.1 ± 11.2	85.0 ± 10.5	88.4 ± 9.16	0.148
N1, %	15.0 ± 8.21	12.5 ± 8.03	11.3 ± 6.18	14.8 ± 9.61	15.4 ± 7.44	19.2 ± 7.78	<0.001
N2, %	43.0 ± 11.1	43.1 ± 12.1	43 ± 9.31	38.1 ± 10.0	42.6 ± 11.9	47.6 ± 11.1	0.002
N3, %	12.1 ± 6.96	15.8 ± 8.56	14.3 ± 7.33	14.0 ± 6.68	11.6 ± 6.19	8.11 ± 5.06	<0.001
REM sleep, %	16.4 ± 6.24	16.1 ± 7.04	17.6 ± 6.72	18.8 ± 6.08	15.6 ± 6.41	14.2 ± 4.52	0.003
OA events, /h	3.72 ± 6.38	0.14 ± 0.53	0.62 ± 1.42	2.10 ± 2.71	3.36 ± 4.37	9.52 ± 9.61	<0.001
Arousal index, /h	35.5 ± 19.9	17.6 ± 13.2	22.7 ± 16.7	29.6 ± 13.5	40.6 ± 14.8	53.1 ± 17.9	<0.001
RAI, /h	28.6 ± 14.9	11.8 ± 9.55	16.4 ± 9.51	24.6 ± 10.8	31.9 ± 9.26	45.5 ± 9.05	<0.001
Snore index, /h	19.6 ± 19.6	6.14 ± 8.51	8.87 ± 16.6	14.9 ± 17.2	27.5 ± 19.7	30.1 ± 17.7	<0.001
Heart rate max, /min	102.0 ± 18.6	104.4 ± 16.7	98.9 ± 16.9	100.5 ± 20.3	100.1 ± 19.5	107.6 ± 17.5	0.044
Arrhythmia, /h	6.70 ± 24.8	2.07 ± 3.22	2.76 ± 9.43	9.76 ± 34.2	11.7 ± 35.7	4.60 ± 13.6	0.620
ChSI, /h	0.17 ± 0.54	0.0 ± 0.0	0.0 ± 0.0	0.02 ± 0.15	0.17 ± 0.42	0.50 ± 0.92	<0.001
SpO_2_ max, %	92.7 ± 2.59	93.6 ± 2.62	93.8 ± 1.45	92.9 ± 2.47	92.8 ± 1.95	91.1 ± 3.34	<0.001
SpO_2_ min, %	79.4 ± 10.7	88.4 ± 5.91	87.3 ± 3.28	84.1 ± 4.74	77.6 ± 8.33	66.9 ± 10.2	<0.001
Systolic BP max, mmHg	148.4 ± 30.6	126.5 ± 36.5	131.3 ± 18.9	142.1 ± 21.8	151.2 ± 26.5	173.9 ± 31.1	<0.001
Diastolic BP max, mmHg	89.4 ± 11.8	89.3 ± 12.7	83.8 ± 11.8	89.6 ± 13.5	91.4 ± 10.7	93.0 ± 9.31	0.001

*p*-values are from one-way ANOVAs. Abbreviations: max, maximum; min, minimum.

**Table 5:** Multiple regression analysis of factors related to AHI-defined OSA severity.

Characteristic	Unstandardized B	Beta	t	*p*	95.0% CI for B	
					Lower	Upper
Sex	−28.6	−0.48	−7.56	<0.001	−36	−21.1
Neck	1.24	0.22	2.4	0.02	0.22	2.27
Waist	0.39	0.24	1.95	0.05	−0.01	0.79
Mallampatti grade	5.78	0.14	2.12	0.03	0.4	11.2
ESS score	−0.52	−0.1	−2.01	0.05	−1.03	−0.01
PSQI score	−1.52	−0.23	−4.67	<0.001	−2.16	−0.88
STOP-BANG score	10.2	0.66	12.2	<0.001	8.56	11.9
AI	0.4	0.26	7.36	<0.001	0.29	0.51
RAI	0.88	0.44	12.2	<0.001	0.74	1.02
Snore index	0.17	0.11	3.33	0.001	0.07	0.27
ChSI	4.13	0.07	2.33	0.021	0.64	7.63
SpO_2_ min	−0.65	−0.23	−5.16	<0.001	−0.89	−0.4
Systolic BP max	0.08	0.08	2.07	0.04	0.004	0.15

## Appendix A

**Table A1:** Multiple regression analysis of factors related to AHI-defined OSA severity.

Characteristic	Unstandardized B	Beta	t	*p*	95.0% CI for B	
					Lower	Upper
Age	0.05	0.02	0.33	0.74	−0.24	0.34
Age group	−0.25	−0.01	−0.15	0.88	−4.49	2.98
Sex	−28.7	−0.48	−7.56	<0.001	−36	−21.1
Education	−1.11	−0.02	−0.37	0.71	−7.04	4.82
Residency	5.87	0.08	1.18	0.24	−3.95	15.7
Employment	0.37	0.01	0.23	0.82	−2.86	3.61
BMI	0.69	0.15	1.28	0.20	−0.37	1.76
Neck	1.24	0.22	2.4	0.02	0.22	2.27
Waist	0.39	0.24	1.95	0.05	−0.004	0.79
Mallampati grade	5.78	0.14	2.12	0.03	0.4	11.2
Tonsil size	1.67	0.04	0.68	0.50	−3.21	6.55
PSQI score	−1.52	−0.23	−4.67	<0.001	−2.16	−0.88
ESS score	−0.52	−0.1	−2.01	0.05	−1.03	−0.01
FOSQ score	0.09	0.05	0.97	0.33	−0.09	0.28
STOP-BANG score	10.2	0.66	12.2	<0.001	8.56	11.9
TST	−0.94	−0.03	−0.58	0.56	−4.09	2.22
Sleep efficiency	−0.56	−0.2	−1.58	0.12	−1.26	0.14
Sleep latency	0.01	0.01	0.29	0.77	−0.07	0.09
Wake	−0.73	−0.26	−1.58	0.12	−1.63	0.18
N1	0.14	0.04	0.43	0.66	−0.48	0.75
N2	−0.09	−0.03	−0.31	0.76	−0.68	0.49
N3	−0.34	−0.08	−1.11	0.27	−0.94	0.26
REM sleep	0.06	0.01	0.18	0.85	−0.61	0.73
Obstructive apnea	0.16	0.03	0.88	0.38	−0.2	0.52
Arousal index	0.4	0.26	7.36	<0.001	0.29	0.51
RAI	0.88	0.44	12.2	<0.001	0.74	1.02
Snore index	0.17	0.11	3.33	0.001	0.07	0.27
Heart rate max	−0.01	−0.01	−0.14	0.89	−0.11	0.09
Arrhythmia	−0.03	−0.03	−0.96	0.34	−0.1	0.04
ChSI	4.13	0.07	2.33	0.02	0.64	7.63
SpO_2_ min	−0.65	−0.23	−5.16	<0.001	−0.89	−0.4
Systoli BP max	0.08	0.08	2.07	0.04	0.01	0.15
